# Investigating the Effects of Transition Metals and Activated Carbon on Hydrogenation Characteristics of Severely Deformed ZK60 Processed by High-Energy Ball Milling

**DOI:** 10.3390/ma17184562

**Published:** 2024-09-17

**Authors:** Aqeel Abbas, Tzu-Chieh Hsu, Jhe-Yi Lin, Hung-Cheng Ho, Kun-Ming Lin, Hsin-Chih Lin

**Affiliations:** 1 Department of Materials Science and Engineering, National Taiwan University, Taipei 10617, Taiwan; engr.aqeel14@gmail.com; 2 Department of Mechanical Engineering, NFC-Institute of Engineering and Fertilizer Research, Faisalabad 38000, Pakistan; 3 Department of Materials Science and Engineering, Feng Chia University, Taichung 40724, Taiwanrooy9638@hotmail.com (J.-Y.L.); kmlin@fcu.edu.tw (K.-M.L.)

**Keywords:** ZK60-activated carbon, high-cycle hydrogen absorption, hydrogen absorption capacity, hydrogen storage materials, transition metals

## Abstract

The synergic effects of activated carbon and transition metals on the hydrogenation characteristics of commercial ZK60 magnesium alloy were investigated. Severe plastic deformation was performed using equal-channel angular pressing with an internal die angle of 120° and preheating at 300 °C. The ZK60 alloy samples were processed for 12 passes using route B_A_. The deformed ZK60 alloy powder was blended with activated carbon and different concentrations of transition metals (Ag, Pd, Co, Ti, V, Ti) using high-energy ball milling for 20 h at a speed of 1725 rpm. The amount of hydrogen absorbed and its kinetics were calculated using Sievert’s apparatus at the higher number of cycles at a 300 °C ab/desorption temperature. The microstructure of the powder was analyzed using an X-ray diffractometer and scanning electron microscope. The results indicated that 5 wt% activated carbon presented the maximum hydrogen absorption capacity of 6.2 wt%. The optimal hydrogen absorption capacities were 7.1 wt%, 6.8 wt%, 6.7 wt%, 6.64 wt%, 6.65 wt%, and 7.06 wt% for 0.5 Ag, 0.3 Co, 0.1 Al, 0.5 Pd, 2 Ti, and 0.5 V, respectively. The hydrogen absorption capacities were reduced by 35.21%, 26.47%, 41.79%, 21.68%, 26.31%, and 26.34% after 100 cycles for 5C0.5Ag, 5C0.3Co, 5C0.1Al, 5C0.5Pd, 2Ti, and 5C0.5V, respectively. Hydrogen absorption kinetics were significantly improved so that more than 90% of hydrogen was absorbed within five minutes.

## 1. Introduction

Fossil fuels pollute nature and have limited sources. The greenhouse effects caused by fossil fuels have also increased the Earth’s temperature [1]. There is a great need to replace fossil fuels with clean and green energy as future energy sources [2]. Hydrogen is an ideal energy carrier, and the development of hydrogen energy focuses on the production, storage, transportation, and application of hydrogen. Among them, storage and transportation are the biggest obstacles to a hydrogen energy economy because hydrogen reacts and burns spontaneously at room temperature [3].

Hydrogen, the most abundant and lightest element, is a colorless, odorless, non-toxic, and extremely flammable diatomic gas. Hydrogen can be stored in high-pressure cylinders and in metals as hydrides. Solid-state hydrogen storage is advantageous over high-pressure storage because of its high energy density, low energy consumption, and high diffusion coefficient [4]. The hydrogen storage mechanism in hydrogen storage alloys involves hydrogen penetrating the crystal lattice and forming metal hydrides. Different metal elements have different affinity for hydrogen. Hydrogen storage alloys can store hydrogen for a long period with low energy loss, avoiding the risk of explosion and high purity [5,6].

Hydrogen storage alloys are metal compounds that can store and release hydrogen. They should have the characteristics of easy activation, high hydrogen storage capacity, moderate hydrogen absorption and desorption reaction pressure, fast hydrogen absorption and desorption rate, long life, and low cost [7,8]. The principle is that hydrogen molecules adsorb on the surface of the alloy under suitable temperature and pressure, and decompose on the surface of the alloy into hydrogen atoms that diffuse into the alloy, react with the alloy atoms to form metal hydrides, and generate heat [9].

Magnesium is one of the lightest metals, with a density of 1.74 g/cm^3^, and is widely used as hydrogen storage material [10]. The theoretical hydrogen storage capacity can reach 7.6 wt%, but its slow hydrogen absorption rate and high ab/desorption temperature are its major disadvantages [11,12]. Magnesium is very active and forms an oxide layer on the surface that hinders hydrogen penetration and reaction between hydrogen and magnesium [13,14]. Mg(OH)_2_ is easily formed on the surface of Mg due to its low corrosion resistance. The hydrogen absorption rate is mainly determined by (a) the rate at which hydrogen molecules on the alloy’s surface dissociate into hydrogen atoms, (b) the ability of hydrogen atoms to penetrate the surface, (c) the ability of hydrogen atoms to diffuse in the metal lattice to form hydrides [4,15].

The main synthesis methods of Mg-based alloys include high-temperature smelting, chemical synthesis, solid-phase diffusion, and mechanical alloying. The hydrogen absorption rate can be improved by enhancing the surface area, which can be increased by nano-structuring the alloys [16,17]. Equal-channel angular pressing (ECAP) is one of the advanced severe plastic deformation techniques used for ultra-fine grain refinement and higher crystal lattice defect density, which consequently enhance hydrogen absorption capacity [18,19,20].

High-energy ball milling (HEBM) is one of the most advanced techniques for alloying hydrogen storage materials. The powder undergoes a lot of severe plastic deformation and fracturing to produce more surfaces for hydrogen absorption. As the ball milling continues, particles become brittle and hardened due to work hardening [21]. The embrittlement and lamellar structures formed during ball milling produce fresh surfaces, increasing the reaction area for the powders. A large amount of strain energy is accumulated through the collisions between the ball and the powder, increasing the local temperature between the ball and the powder, which is beneficial for accelerating the diffusion and synthesis between the powder. It has been reported that alloy processed through ECAP and ball milling exhibit the maximum hydrogen absorption/desorption rates at 300 °C [22].

Alloying magnesium alloy with transition metals (TMs) reduces the kinetic barriers and facilitates the reaction rate. Transition metals (Al, Pd, Co, Ag, Ti, and V) are excellent catalysts and improve the de/hydriding kinetics of magnesium at higher temperatures (>573 K). These transition metals weaken the Mg-H bond and decrease the de/hydrogenation temperatures [23,24,25,26]. The mechanical milling of these transition metals could protect from oxidation and therefore improve hydrogen absorption. The main advantages of transition metals (Al, Pd, Co, Ag, Ti, and V) as hydrogen storage materials are their high volumetric densities (>100 KgH_2_/m^3^) and the ability to operate at ambient temperature and pressure. These transition metals were selected because of their low thermodynamic stability, higher structural defects, high valence state, and high interaction energy.

Yongqing Wang et al. [27] summarized the catalytic effects of transition metals and rare-earth metals on magnesium alloys: they lower the activation energy and increase the surface area for an effective hydrogen absorption rate. D. Chen et al. [28] ball-milled Mg with 5 wt% of multi-walled carbon nanotubes (MWCNTs) for hydrogenation and achieved 6.08% hydrogen absorption capacity. Yongqing [27] also investigated the effects of Al and Ti on magnesium and observed 4.20 wt% hydrogen capacity. The effects of Ni and Co on hydrogen absorption capacity of AZ61 was investigated by Peter Mose et al. [29] and they obtained 4.5 wt% in AZ61-Ni and 5.13 wt% in AZ61-Ni + Co. Huang et al. [30] investigated the hydrogen absorption capacity of AZ31 and AZ91 and found 6.0 wt% in AZ31 and 6.5 wt% in AZ91, indicating that Al is a better catalyst for hydrogen absorption. Hydrogen absorption capacities of magnesium with different additives and temperatures have been summarized in Table 1. It can be concurred that hydrogen absorption capacity is significantly influenced by different additive catalysts and absorption temperatures. Transition metals such as Ti, Vi, and Ni are efficient catalysts for improving the hydrogen absorption capacity of magnesium.

The synergetic effects of activated carbon and transition metals have further enhanced the hydrogen storage properties of Mg-based materials [23,38]. The hydrogenation capacity of a hydrogen storage alloy will reduce with the increasing number of hydrogen absorption cycles and desorption. Most of the researchers have been focusing on the low frequency of absorption and desorption cycles. The effects of severely deformed ZK60 with activated carbon and transition metals at a higher number of cycles have not been addressed yet. The synergetic effects of activated carbon and different concentrations of transition metals at a higher number of cycles have been discussed.

This research aimed to investigate the effects of activated carbon and transition metals on the hydrogenation characteristics of ZK60 alloy. ECAP was used to deform the ZK60 alloy and blend it with different transition metals along with 5 wt% activated carbon using high-energy ball milling. The hydrogen absorption performance at a higher number of hydrogenation cycles was analyzed.

## 2. Experimental Procedures and Methods

ZK60 alloy with a composition of Mg-5.7 wt%Zn–0.6 wt%Zr provided by Kuangyue Co., Ltd., Taiwan, China. was used as the base material to investigate hydrogen absorption performance. The samples, with dimensions of 11 mm × 11 mm, were prepared for ECAP. The ZK60 alloy was severely deformed via equal-channel angular pressing (ECAP) with route B_A_ (samples are rotated by 90° in an alternative direction after each pass) at an internal die angle of 120°, preheated at 300 °C, and extruded at a rate of 0.5 mm/min. A total of 12 passes were used to extrude the samples under the same operating conditions.

Mechanical filing was used to remove the chips from severely deformed ZK60. The chips were blended with 5 wt% activated carbon and different concentrations of transition metals (Al, Pd, Co, Ag, Ti, and V) supplied by First Chemical Co., Ltd., Xuzhou, China. using high-energy ball milling (Spex8000-D). All materials were 99.98% pure. The HEBM was performed with a ball-to-powder ratio of 5:1 and speed of 1725 rpm for 20 h. The process was stopped for 15 min after each 30 min to avoid high-temperature effects. The whole process was carried out in an argon atmosphere. Hydrogen absorption characteristics were analyzed using a commercial Sievert’s apparatus. The sample chamber was maintained at a specific temperature by constant temperature heating, and impure gases were extracted by creating a vacuum up to 3 × 10^−3^ torr. After this, hydrogen with a purity of 99.99% was supplied to investigate hydrogen absorption and reaction. A computer program was used to record the change in pressure, volume, and temperature. The hydrogenation capacity of the alloy was measured by the theoretical density of the hydrogen and alloy weight. The alloys were activated at 400 °C for 10 h before hydrogenation to remove the passivation layer of oxides. Hydrogen was supplied at 40atm pressure at 300 °C for hydrogenation, and changes in pressure with time were observed. The rate of hydrogen absorption and maximum hydrogen absorption were measured over a period of 1 h.

An X-ray diffractometer (MAC-MXP3) operated at 50 kV and 300 mA with a CuK light source having a wavelength of 1.5406 Å, and a diffraction range extending from 20° to 80° at a scan rate of 4°/min to analyze the phase composition and crystalline structure. A scanning electron microscope (SEM) was used to observe the powder morphology of the alloys.

## 3. Results and Discussion

### 3.1. Hydrogenation Characteristics

**Figure 1:**
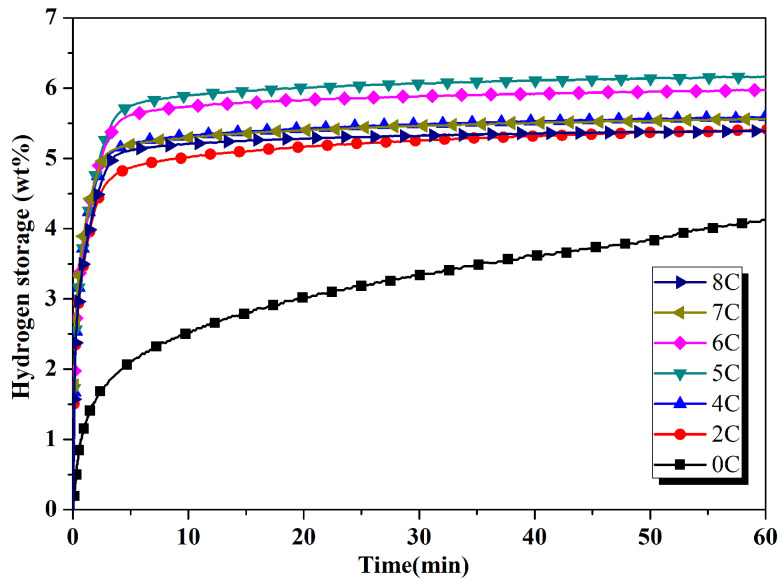
Hydrogen absorption performance of ZK60 alloy with different proportion of activated carbon.

Figure 1 presents the hydrogenation characteristics of ECAPed ZK60 with different concentrations of activated carbon and it can be observed that the maximum hydrogen (4.06 wt%) is absorbed in 1 h in the ECAPed ZK60 alloy. The hydrogen absorption rate is slow and does not reach the saturated hydrogen absorption capacity. The hydrogen absorption capacity of the ECAPed ZK60 alloy has been improved by the addition of activated carbon (C). The hydrogen absorption capacity increases with the increase in concentration of activated carbon and it reaches a maximum value of 6.24 wt% at 5 wt% addition. The hydrogen absorption curve is almost horizontal, indicating that it is close to saturation hydrogen absorption (Figure 1). The absorption capacity is reduced with further increase in the concentration of activated carbon. Huang et al. used [30] AZ31 and AZ91 for hydrogenation investigation after the ECAP and ball-milling process. They found 5.4 wt% in AZ31 and 6.04 wt% in AZ91 magnesium alloy.

**Figure 2:**
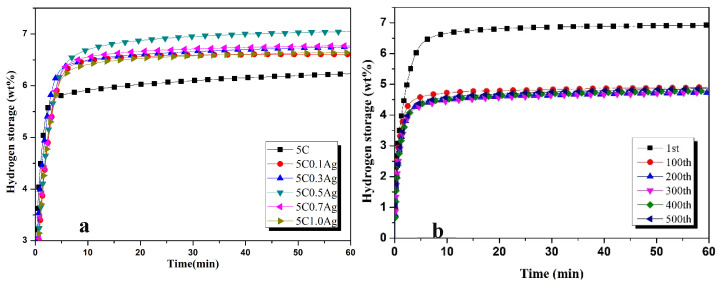
Hydrogen absorption performance of Zk60 with (**a**) 5C+ different additives of Ag and (**b**) 5C + 0.5Ag at higher number of cycles.

ZK60 with 5 wt% of activated carbon was selected to investigate the effects of a different concentration of different transition metals. Figure 2a presents the hydrogenation characteristics of ZK60 alloy with 5 wt%C and different proportions of Ag (wt%) at 300 °C. It can be observed that the saturated hydrogen absorption capacity of ECAPed ZK60 improves extensively with the addition of Ag because of its excellent catalytic properties. Thus, the addition of a very small amount of Ag improves the hydrogenation capacity effectively. The addition of 0.1 wt%Ag improves the hydrogenation capacity to 6.6 wt%. The hydrogen absorption capacity is enhanced with the increase in concentration of Ag and reaches a maximum value of 7.1 wt% with the addition of 0.5 wt%Ag. Further increase in concentration of Ag leads to a decrease in hydrogenation capacity. It has been reported [39,40] that Ag has excellent conductivity and is homogeneously distributed during the ball-milling process. Thus, it has the ability to enhance hydrogen absorption capacity to maximum level. It can quickly conduct the required heat energy efficiently during hydrogen absorption and release. Moreover, it increases grain boundaries and promotes reaction kinetics [24].

The ZK60 alloy powder with 5wt% activated carbon and 0.5 wt%Ag (hereafter 0.5Ag) was selected for hydrogen absorption at the higher number of cycles and the results are presented in Figure 2b. The first hydrogen absorption of 0.5Ag is 6.8 wt% and is reduced to 4.5 wt% after the 100th absorption cycle. The hydrogen absorption is reduced by 33.8% after the 100th cycle. The hydrogenation capacity remains the same after the 100th cycle. Ag can promote H_2_→2H on the hydrogen storage material’s surface and can quickly form a hydride with Mg. Ag can also catalyze the reaction of MgH_2_→Mg + 2H during the hydrogen release process, which allows hydrogen atoms to diffuse rapidly through the surface of the alloy and reduce to hydrogen molecules. Therefore, silver can ensure that ZK60 hydrogen storage materials have good cycling properties [25].

**Figure 3:**
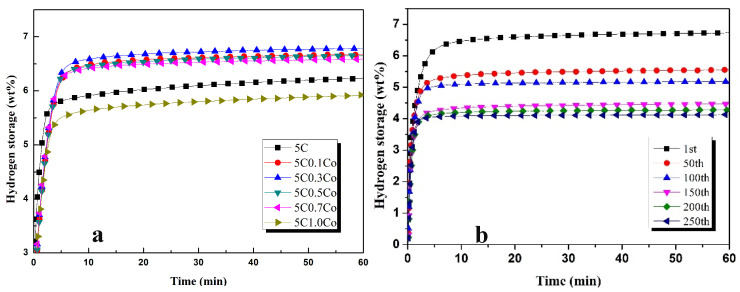
Hydrogen absorption performance of Zk60 with (**a**) 5C + different additives of Co and (**b**) 5C + 0.3Co at higher number of cycles.

Figure 3a presents the hydrogen absorption characteristics of ZK60 with different proportions of Co. It can be observed that when the concentration of Co is increased from 0.1 wt% to 0.3 wt%, hydrogen absorption capacity is increased from 6.5 wt% to 6.8 wt%. The further addition of 0.5 wt% and 0.7 wt% reduces the hydrogen absorption capacity to 6.45 wt% and 6.4 wt%, respectively. The addition of 1 wt%Co drops the hydrogen absorption to 5.5 wt% after 1 h of hydrogenation. The solid solution formed by the addition of Co with ZK60 and activated carbon is the endothermic process, which enhances the hydrogen absorption/desorption process [41].

The effects of the higher number of cycles on the hydrogenation characteristics of ZK60 with 5 wt% activated carbon with 0.3 wt% of Co are shown in Figure 3b. The hydrogen absorption capacity in the first cycle with 0.3 wt%Co addition is observed as 6.4 wt%, which reduces to 5.3 wt% after 50 cycles. The hydrogen absorption capacity is reduced to 5 wt% after 100 cycles. The hydrogen absorption capacity is reduced by 25% after the first 50 cycles and 2% after the next 50 cycles. The hydrogen absorption capacity drops to 4.1 wt% after 150 cycles, which is a 2% reduction after 100 cycles. A very small reduction in hydrogen absorption capacity (3.9 wt%) is observed after the next 50 cycles [42,43].

**Figure 4:**
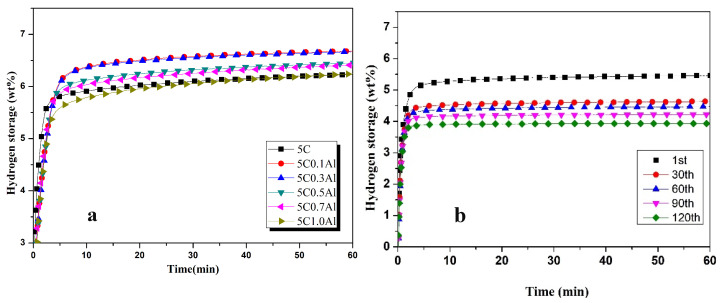
Hydrogen absorption performance of Zk60 with (**a**) 5C + different additives of Al and (**b**) 5C + 0.3Al at higher number of cycles.

The effects of different proportions of Al (wt%) on hydrogen absorption are presented in Figure 4a. It can be seen that the addition of a small amount of Al increases the hydrogen absorption capacity. The addition of 0.1 wt% Al increases the hydrogen absorption capacity to 6.7 wt% after 1 h of the hydrogenation process. The addition of 0.3 wt% Al has almost the same effects as 0.1 wt%Al. The Al concentration higher than 0.3 wt% decreases the hydrogen absorption capacity after 1 h of hydrogenation. The excessive amount of Al causes a reduction of alloy atoms and the cell gap becomes smaller, reducing hydrogen penetration, leading to a decrease in hydrogen absorption capacity. The addition of Al in Mg enhances the alloy’s stability and weakens the stability of hydrides, which improve the hydrogen release performance of the Mg-Al system [44]. It is less likely to produce irreversible stable hydrides. Al is cheap, environment-friendly, and very effective at reducing hydride stability. The addition of a small amount of Al will improve the hydrogen absorption characteristics. Andreasen [34] investigated the effects of Al and Ti on hydrogenation characteristics of AZ61 and found 4.06 wt% hydrogen absorption capacity.

The higher cycles have uniform reduction effects at the hydrogenation capacity of ZK60 with 0.3 wt%Al, as indicated by Figure 4b. The hydrogen absorption capacity during the first cycle (5.43 wt%) is reduced to 4.44 wt% after 30 cycles. A uniform reduction of 2% is observed after every 30 cycles. The amount of hydrogen absorbed by adding 0.3 wt%Al decreases rapidly as the number of cycles increases, and there is no obvious trend of slowing down.

**Figure 5:**
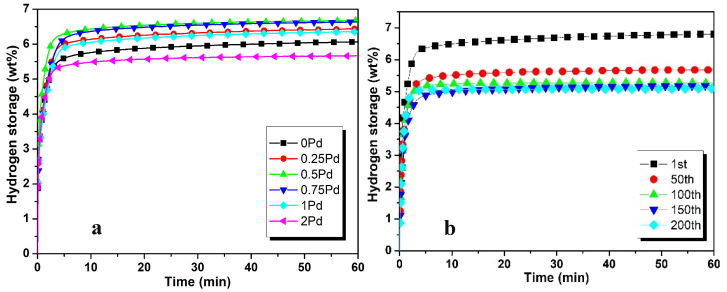
Hydrogen absorption performance of Zk60 with (**a**) 5C + different additives of Pd and (**b**) 5C + 0.5Pd at higher number of cycles.

The hydrogenation characteristics of ZK60 with different concentrations of Pd are presented in Figure 5a. It can be seen that maximum hydrogenation capacity (6.7 wt%) is reached with 0.5 wt%Pd addition. Further addition of Pd reduces the hydrogen absorption capacity, which drops to 5.2% with 2 wt%Pd. The hydrogen absorption of ZK60 with 0.5 wt%Pd at a higher cycle is presented in Figure 5b. The hydrogenation capacity of ZK60 alloy with 0.5 wt%Pd addition can be observed as 6.8 wt% at the first cycle, which is reduced to 5.6 wt% by the 50th absorption/desorption cycle. Hydrogen absorption capacity is reduced by 17.6 wt% after 50 hydrogenation cycles. The hydrogen absorption capacity of ZK60 with 5 wt%C and 0.5 wt%Pd has reduced to 4.7 wt% at the 200th cycle, which is a 30.8% reduction compared with the first cycle. Therefore, from the 50th cycle, the amount of hydrogen absorption decreases by only 3.0%. It shows that the addition of 0.5Pd is very good for improving cyclic hydrogen absorption and desorption performance.

**Figure 6:**
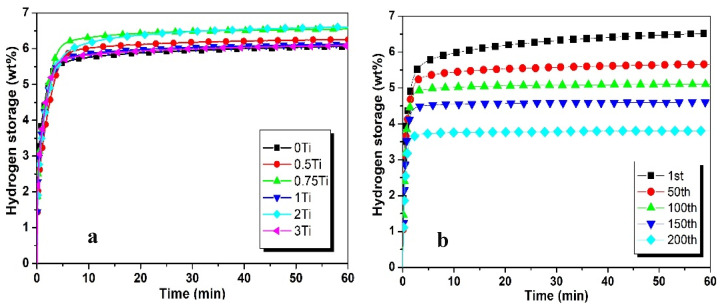
Hydrogen absorption performance of Zk60 with (**a**) 5C + different additives of Ti and (**b**) 5C + 2Ti at higher number of cycles.

**Figure 7:**
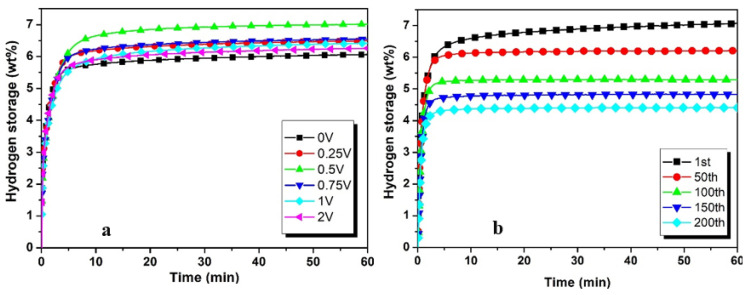
Hydrogen absorption performance of Zk60 with (**a**) 5C + different additives of V and (**b**) 5C + 0.5V at higher number of cycles.

The maximum hydrogen absorption (6.5 wt%) with 2 wt% Ti addition is indicated by Figure 6a. The 0.75Ti and 2 wt%Ti concentrations result in the same hydrogen absorption capacity after 1 h of the hydrogenation process. The other Ti concentrations have dropped the hydrogenation capacity but are almost the same after 1 h. A similar trend in hydrogen absorption capacities is observed when vanadium’s different concentration is added in ZK60, as shown in Figure 7a. The increase in vanadium concentration enhances hydrogen absorption. It reaches the optimal value of 6.7 wt% with 0.5 wt%V, and further addition causes a reduction in hydrogen absorption capacity, reaching 5.7 wt% with 2 wt% addition of vanadium [45].

Vanadium and titanium exhibit very similar hydrogenation patterns at a higher number of cycles, as indicated in Figure 6b and Figure 7b. Hydrogen absorption capacity is reduced by 17% after the first 50 cycles and 18% after the next 50 cycles. After 100 cycles, hydrogen absorption is reduced by 3% after each 50 cycles.

The increase in the number of cycles decreases the hydrogen absorption performance of ZK60 alloy. The addition of higher contents of transition metals (Ag, Pd, Co, Ti, V, Al) expands the unit cell volume, reducing the expansion/contraction rate of the hydrogen storage alloy (ZK60) during the hydrogen de/absorption process by resisting micronization. Powder micronization is a very serious agglomeration phenomenon, which leads to a reduction in hydrogen absorption at higher contents. The small amount of transition metal additives increases the volume expansion rate to 20~30%, which leads to powder breaks that enhance hydrogen absorption significantly. The continuous hydrogen absorption and desorption cycle causes the powder to continue to be broken and micronized [24].

The decrease in hydrogen absorption at a higher number of cycles can be attributed to the fact that many stable hydrides are formed that cannot be dissociated into Mg and 2H. Another reason can be the slow hydrogen release rate. Adding a small concentration of transition metals acts as a catalyst that removes impurities and oxides on hydrogen storage materials’ surfaces. It can even reduce the activation temperature and time [9]. On the other hand, hydrogen has good permeability on these transition metal membranes, and the diffusion rate is fast. The film established can also block the entry of large molecules such as oxygen [46].

Table 2 shows the amount of hydrogen absorbed in 60 min with different proportions of Ag (wt%). The amount of hydrogen absorbed per minute in the first five minutes and its percentage has been calculated. It can be seen from Table 2 that the hydrogen absorption rate will decrease in the first 2 min when Ag is added. A total of 75.8% of hydrogen is absorbed in the first 2 min and 91.93% in 5 min of hydrogenation when Ag is not added. The addition of 0.3 wt% of Ag increases the hydrogen kinetics so that 77.61% of hydrogen is absorbed in the first 2 min and 95.52% in 5 min of hydrogenation. The addition of Co is significant in improving the hydrogen kinetics, as is clear from Table 2. The percentage of hydrogen absorbed in the first 2 min is decreased when Co is added but is increased in 5 min. The increase in the percentage of hydrogen absorbed in 5 min indicates that Co has increased the electrochemical performance of ZK60 alloy.

The hydrogen absorbed in 60 min, and its percentage absorbed at 2 min and 5 min with different Al contents, is about 91.93%, as shown in Table 2. It can be observed that hydrogen absorption increases in 5 min as Al contents are increased, showing that the addition of Al increases the kinetics of hydrogenation in 5 min.

The hydrogen absorption capacity in 5 min increases with the increase in Pd contents and percentage of hydrogen absorbed, as indicated by Table 2. The addition of 2 wt% Pb leads to 86.94% hydrogen absorption in 2 min and 95.06% in 5 min. Maximum hydrogen absorption within 5 min is observed when 2 wt%Ti is added, and 97.71% absorption takes place in 5 min before saturation (Table 2). Hydrogen absorption in 5 min is increased as Ti contents are increased, indicating that Ti addition has increased the absorption kinetics. A similar hydrogen absorption phenomenon is observed when a small amount of vanadium is added (Table 2). The hydrogen absorbed in 5 min is increased with the increase in vanadium. The percentage of hydrogen absorbed in 5 min is less than when no vanadium is added, except with 1 wt%V. The amount of hydrogen absorbed within the period of 5 min is increased with an increase in vanadium concentration.

Hydrogen absorption in metals involves many steps, i.e., surface penetration, chemisorption, physisorption, diffusion, and hydride formation. The mechanism differs according to the amount of catalyst.

The hydrogenation mechanism of magnesium reinforced with transition metals reveals that Mg2TsM nano particles are excellent catalysts. The amount, uniform distribution, and intrinsic catalytic properties of transition metals result in cycle efficiency. The carbon attached to the surface of transition metals inhibits the aggregation of the catalyst and prompts the de/hydrogenation process.

There are several solid-state hydrogenation mechanism models, including the reaction order model, diffusion mobility model, geometric contraction model, and nucleation and growth model, that are used to elucidate diffusion kinetics. The reaction rate is described by the following Equation (1)

dα⁄dt = kf(α) (1)

where f(α) is function-dependent on the reaction mechanism, k is the reaction rate constant influenced by temperature, and α is the reaction extent.

The hydrogenation mechanism shows that Mg_2_TsM and Mg_2_TsMH_4_ are active catalytic species leading to easier H dissociation and nucleation. The carbon on the surface of catalyst plays a significant role in hindering the growth of the catalyst during the cycling process [47].

There are many factors that affect the hydrogenation of magnesium and its alloys: (a) A higher number of structural defects leads to higher catalytic efficiency; (b) Low thermodynamic stability is needed to allow chemical interaction (c) high chemical stability should (d) high valance state to ensure a catalytic effect; (e) high interaction energy with hydrogen?

Hydrogen molecules are dissociated from a transition metal ion catalyst with the formation of a hydrogen transition metal bond. Transition metal ions have multiple valance states and this ability allows them to easily dissociate hydrogen molecules and pass them to the nearest magnesium atom. The transition metal ions can then dissociate other hydrogen molecules from the gaseous phase. The slow diffusion rate decelerates the process and therefore limits the kinetics for catalyzed nanocrystalline. Nevertheless, higher catalytic activity improves the chemical potential of hydrogen in the hydride phase and accelerates the diffusion rate. Therefore, the diffusion rate is also influenced by the choice of catalyst.

Mg-H bonds are chemically unstable, leading to easier dissociation of magnesium hydrides, since diffusion of hydrogen through magnesium is relatively fast and hydrogen atoms reach the surface rapidly. The transition metal ion catalyst then forms a bond with the hydrogen atoms and acts as an intermediate state, which allows easier transition of the hydrogen atoms towards a molecular state.

The transition metal ions play a catalytic role and their ability to form hydrogen bonds with different stoichiometries provides a faster route for dissociation of hydrogen atoms. The anions, such as oxygen, halogens, and carbons, change the electronic structure of transition metal ions and are essential for their catalytic behavior. The interaction with magnesium and defects in the catalyst’s surface alter the electronic structure and influence the ability of transition metal ions to form hydrogen bonds.

The synergic effects of activated carbon and transition metals can be observed in Figure 1, Figure 2, Figure 3, Figure 4, Figure 5, Figure 6 and Figure 7. The hydrogen absorption capacities and kinetics are increased, positively influencing the synergic effects. The amount of transition metal plays a significant role in hydrogen absorption. An increase in concentration of transition metal improves hydrogen absorption capacity, but after a certain level, capacity decreases. Higher concentration reduces the penetration rate and physisorption. The synergic effects of activated carbon and transition metals have the negative effect of cyclic hydrogenation. The higher number of cycles leads to reduction in hydrogen absorption. Transition metal ions are excellent catalysts for hydrogenation, and activated carbon atoms attached to the surface of TMs hinder the cyclic growth of the catalyst, thereby reducing hydrogen absorption capacity after each cycle.

**Figure 8:**
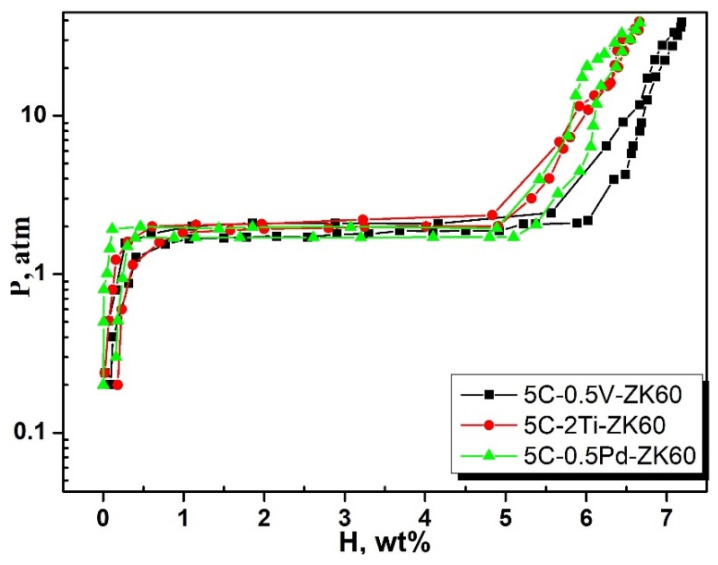
P-C-T curves for ZK60 alloy with the powders of different elements.

The P-C-T curve measurements were carried out to understand the hysteresis behavior of the hydrogen storage alloy. Three powders were selected for further P-C-T curve measurements. The samples were activated for 10 h, and hydrogen absorption and desorption temperatures were set to 400 °C/300 °C. Figure 8 shows the P-C-T curves of ZK60 alloy with different additives.

The existence of a single pressure plateau can been observed. The plateau area’s slope is related to the energy of the hydrogen atoms occupying the interstitial positions of the powder. Hydrogen enters gap positions with lower energy during diffusion and lattice gap positions with high energy. The equilibrium pressure in absorption and desorption in the plateau area represents the maximum reversible hydrogen absorption capacity. The gradient and hysteresis are exhibited by hydrogenation/dehydrogenation. A single plateau with very small hysteresis can be observed, which represents the reaction between Mg/MgH_2_. The very small hysteresis is attributable to the reversible reaction process.

### 3.2. Microstructural Characterization

**Figure 9:**
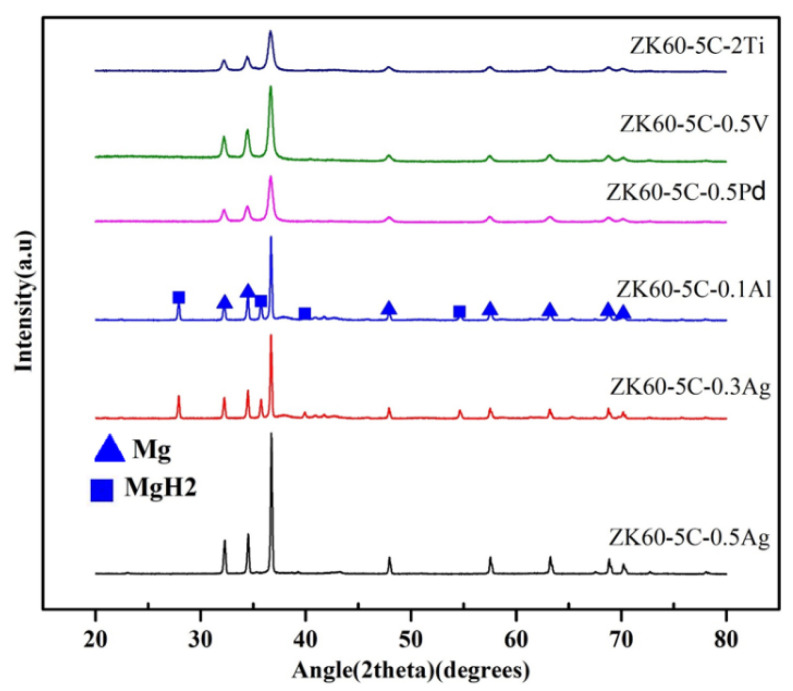
XRD analysis of ZK60 with different additives.

Figure 9 shows the XRD patterns for the hydrogen storage alloy (ZK60) with different additives after high-cycle hydrogen release. The alloys with maximum hydrogen absorption capacity were selected for XRD analysis. It can be observed that the addition of Pd, V, Ti, and Co has almost no peak of MgH_2_. This means that after high-cycle hydrogen absorption and desorption of ZK60 with these additives, there is no obvious stable hydride remaining and the cycle performance is very good. The diffraction peak intensities are reduced, whereas FWHM is increased due to reduction in crystallite size. The residual Mg powder forms MgH_2_ and more peaks are visible in XRD analysis. The presence of tiny MgH_2_ peaks is due to two possibilities. The first is that there is a trace of stable hydride remaining in the alloy that cannot be dissociated into Mg and 2H [25]. The second possibility is that the hydrogen release is too slow. The hydrogen release time of one hour cannot completely dissociate MgH_2_ into Mg and 2H. This also explains why the amount of hydrogen absorption will gradually decrease as the number of cycles increases, and its cycle performance is better. The peak intensity of MgH_2_ with Al is higher than the others. It also shows that the cycle properties of Al addition are the worst.

**Figure 10:**
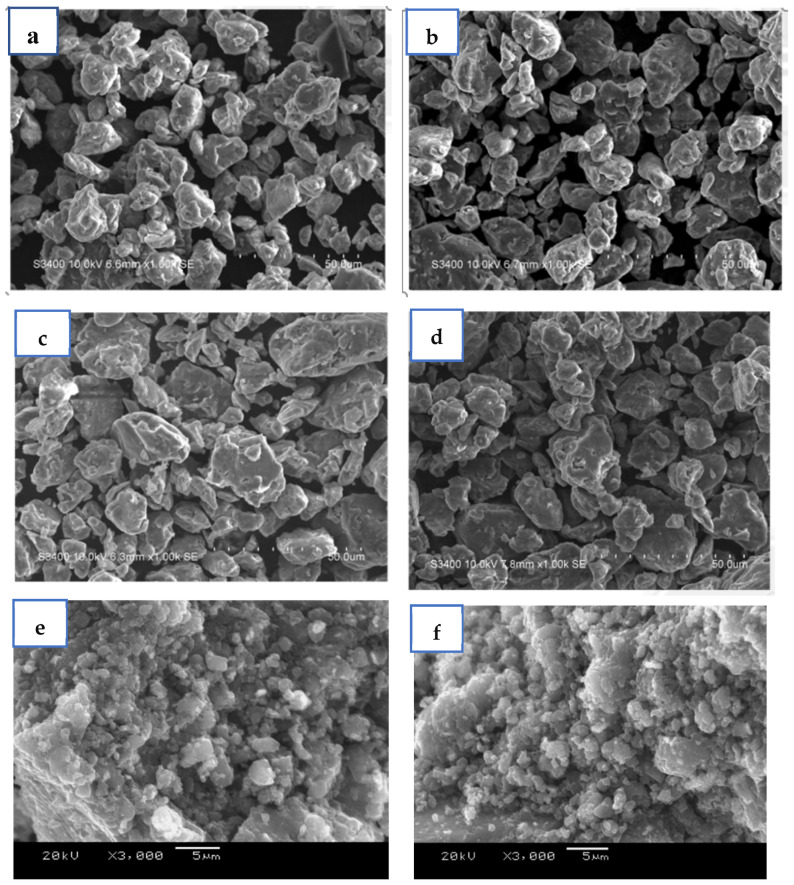
FESEM images of ZK60-5C with (**a**) 0.5Ag (**b**) 0.3Co (**c**) 0.3Al (**d**) 0.5Pd (**e**) 2Ti (**f**) 0.5V after dehydrogenation.

Figure 10 shows the SEM images of hydrogen storage alloy powder with different additives after dehydrogenation. There is no significant difference in surface morphology and approximate particle size varies from 20 μm to 70 μm. The powder sample of 2Ti and 0.5V (Figure 10d,e) is provided at higher magnification for better understanding of particle distribution and their bonding. The uniformity of nanocrystalline grains significantly influences the interface migration and leads to 20~30% volume expansion, which consequently breaks the powder [48,49]. The higher hydrogen absorption causes the higher degree of micronization via expansion, which consequently leads to decrease in particle size. The continuous hydrogen absorption/desorption leads to continuous micronization. The degree of micronization decreases with the increase in number of cycles of hydrogenation, as the expansion rate is slow, which causes a reduction in hydrogen absorption capacity at a higher number of cycles. The equal particle size of all the powders indicates that all samples have almost the same hydrogen absorption capacity initially and vary at a higher number of cycles as the expansion rate varies.

## 4. Conclusions

In summary, ZK60 alloy was deformed using 12 passes of ECAP and blended with different contents of transition metals (Pd, Ag, Co, Ti, V, and Al) using HEBM. The effects of transition metal addition and the number of high cycles on hydrogen absorption characteristics were investigated. The results were as follows:

1. The addition of 5 wt% activated carbon increased hydrogen absorption capacity from 4.06 wt% to the maximum level of 6.2 wt%.
2. The addition of different contents of transition metals increased the hydrogen absorption capacity of ZK60 + 5C, and maximum hydrogen capacities in the first cycle after 1 h were observed as 7.1 wt%, 6.8 wt%, 6.7 wt%, 6.64 wt%, 6.65 wt%, and 7.06 wt% for 0.5Ag, 0.3Co, 0.1Al, 0.5Pd, 2Ti, and 0.5V, respectively.
3. Hydrogen absorption capacities decreased by 35.21%, 26.47%, 41.79%, 21.68%, 26.31%, and 26.34% for 0.5Ag, 0.3Co, 0.1Al, 0.5Pd, 2Ti, and 0.5V, respectively, after 100 cycles.
4. Hydrogen absorption in ZK60 with 5C0.5Ag and 5C0.5Pd remained the same in the cycles higher than 100 but decreased in all other samples.
5. The hydrogen absorption percentage in the first five minutes was higher than 90% in all samples and effectively improved the hydrogen absorption characteristics.
6. There was no obvious difference in microstructure between samples with different additives, and hydrogen was completely desorbed in all samples except 5C0.1Al and 5C0.3Ag at higher cycles.

## Figures and Tables

**Table 1:** Hydrogen absorption capacities of Mg with different additives and under different conditions.

Alloy	Absorption Temperature (°C)	Hydrogen Absorption Capacity (wt%)	References
Mg-Ni	300	3.6	[31]
Mg-5 wt%Ti	320	6.0	[32]
Mg-10 wt%V	250	6.5	[33]
Mg-Al-Ti	360	4.28	[34]
AZ61	375	3.9	[27]
AZ61-Ni + Co	375	5.13	[29]
ZK60-Graphene + (1 mL) Toluene	320	6.36	[35]
ZK60-Graphene +(2 mL) Cyclohexane	320	6.77	[35]
ZK60-5C + 2V	350	6.7	[36]
ZK60-5 wt% activated carbon	300	6.6	[37]

**Table 2:** Hydrogen absorption with activated carbon and different transition metals.

Sample	60 min (wt%)	2nd Min		5th Min	
		wt%	%	wt%	%
ZK60 + C	6.2	4.7	75.8	5.7	91.93
0.1 Ag	6.6	4.6	69.69	6.2	93.93
0.3 Ag	6.7	5.2	77.61	6.4	95.52
0.5 Ag	7.1	4.8	67.6	6.4	90.14
0.7 Ag	6.8	4.7	67.64	5.3	67.64
1.0 Ag	6.7	4.6	68.65	6.2	92.53
0.1 Co	6.7	4.7	70.14	6.3	94.02
0.3 Co	6.8	4.8	70.58	6.4	94.11
0.5 Co	6.7	4.7	70.14	6.3	94.02
0.7 Co	6.6	4.8	72.72	6.2	93.93
1.0 Co	5.9	4.6	77.96	5.6	94.91
0.1 Al	6.7	4.7	70.14	6.2	92.53
0.3 Al	6.7	4.6	68.65	6.2	92.53
0.5 Al	6.5	4.7	72.3	6.0	92.3
0.7 Al	6.4	4.7	73.43	6.3	98.43
1Al	6.3	4.5	71.42	5.6	88.88
0.25Pd	6.44	5.09	79.03	6.01	93.32
0.5Pd	6.64	4.97	74.84	6.16	92.77
0.75Pd	6.56	5.5	83.84	6.13	93.44
1Pd	6.36	4.79	75.31	5.91	92.92
2Pd	5.67	4.93	86.94	5.39	95.06
0.5Ti	6.25	4.82	77.12	5.7	91.2
0.75Ti	6.55	4.82	73.85	6.11	93.28
1Ti	6.57	4.9	74.58	6.42	97.71
2Ti	6.65	4.57	68.72	5.79	87.06
3Ti	6.09	4.71	77.33	5.68	93.26
0.25V	6.50	4.81	74	5.99	92.15
0.5V	7.06	4.71	66.71	6.18	88.15
0.75V	6.72	4.62	68.75	6.0	89.28
1V	6.47	4.74	73.26	6.02	93.04
2V	6.31	4.8	76.06	5.83	92.39

## Data Availability

The raw data supporting the conclusions of this article will be made available by the authors on request.
